# Laryngeal Chondrosarcoma of the Thyroid Cartilage

**DOI:** 10.5146/tjpath.2020.01509

**Published:** 2021-05-15

**Authors:** Selma Erdoğan Düzcü, Zeliha Coşgun, Hesna Müzeyyen Astarcı

**Affiliations:** Department of Pathology, Bolu Abant Izzet Baysal University, Faculty of Medicine, Bolu, Turkey; Department of Radiology, Bolu Abant Izzet Baysal University, Faculty of Medicine, Bolu, Turkey

**Keywords:** Chondrosarcoma, Larynx, Thyroid cartilage

## Abstract

Laryngeal chondrosarcoma is rare and accounts for 0.2% of all larynx malignancies. Although chondrosarcoma is the most common sarcoma seen in the larynx, laryngeal involvement by cartilage tumors is rare. In this article, we aimed to present the differential diagnosis of chondrosarcoma located in the thyroid cartilage, which is a rare site, in a 75-year-old male patient. The patient underwent total laryngectomy by the otolaryngology department. The macroscopy of the laryngectomy material sent to the pathology laboratory revealed a 3x2 cm tumor with a polypoid extension to the lumen from the bottom of the right vocal cord. Although clinical and radiological findings are important in the diagnosis, the definite diagnosis is based on the pathological examination. It is especially important to differentiate the lesion from chondromas.

## INTRODUCTION

Laryngeal chondrosarcoma is rare and accounts for 0.2% of all larynx malignancies (1). Although chondrosarcoma is the most common sarcoma seen in the larynx, laryngeal involvement of cartilage tumors is rare (2,3). Although it mostly develops from the cricoid cartilage, it may also develop from the epiglottis, and the thyroid and arytenoid cartilage (1,4). 20% of the cases develop in the thyroid cartilage (5).

The most common symptom is hoarseness and may come with complaints of difficulty breathing, dysphagia, and hemoptysis (1,6). Tumors located in the thyroid cartilage may especially present with painless mass complaints (7,8). Since the symptoms are similar to those of asthma and laryngitis, its diagnosis requires care in elderly patients (4).

In this article, we aimed to present the differential diagnosis of chondrosarcoma located in the thyroid cartilage, which is a rare site, in a 75-year-old male patient.

## CASE REPORT

A 75-year-old male patient was admitted to the chest diseases department at an external center with the complaint of shortness of breath lasting for three weeks. He was referred to the ear, nose and throat diseases clinic by chest diseases clinic, considering that the patient’s complaint might have been caused by a thyroid disease. Neck laryngography performed on laryngoscopy at the subglottic level reported a hypoechoic nodule of 4.5 x 5 cm size with coarse calcifications that covered a large part of the right lobe of the thyroid gland and had an intrathoracic extension. On thyroid scintigraphy, there were nodules that filled the entire right lobe with a hypoactive appearance in the lower part of the left thyroid lobe, and the appearance was interpreted as a multinodular hyperplasic thyroid gland. On neck MR, there was a nodule of 49x50 mm size with contrast enhancement that created partial obliteration to the trachea by filling the entire thyroid gland, and histopathological correlation was recommended to exclude malignancy.

As a result of these examinations, thyroidectomy and excision of the mass were performed by the otorhinolaryngology diseases clinic, and a temporary tracheostomy was opened. On the 15th day, the tracheostomy was closed. The surgical material sent to the pathology department at the external center was reported as a grade 2 chondrosarcoma invading the thyroid tissue.

PET-CT was taken one month later. F-18 FDG involvement was more pronounced in the non-homogeneous periphery of the lesion, which extended to the thyroid tissue in the neck, and narrowed the air column while destroying the larynx on the right side, while its borders could not be clearly evaluated (SUV max 5.2). Metabolic activity was observed in the lesion described in right thyroid cartilage.

**Figure 1:**
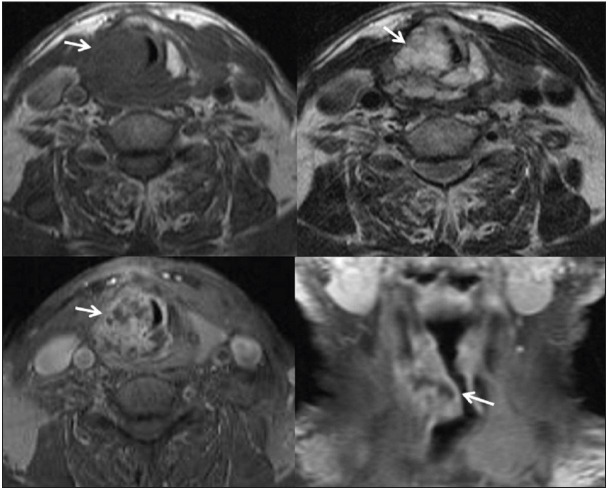
In axial and coronal MRI images, a malignant mass invading thyroid, arytenoid and cricoid cartilages (arrows).

**Figure 2:**
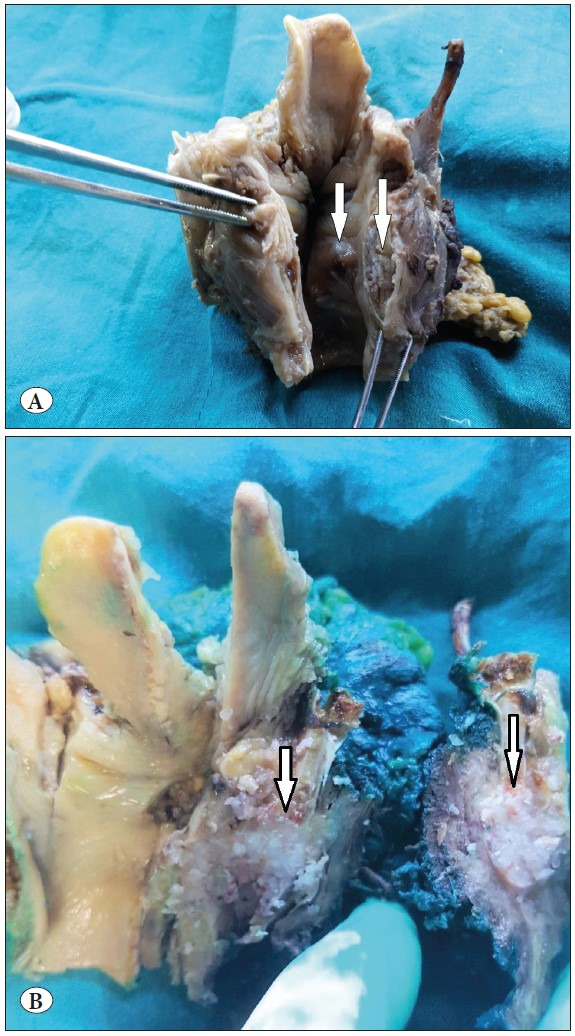
**A)** Macroscopy of the laryngectomy with a 3x2 cm tumor (arrows). **B)** The tumor was located in the thyroid cartilage (arrows).

**Figure 3:**
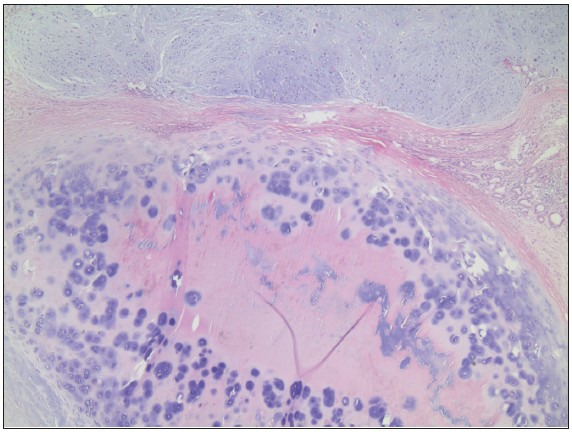
Chondrosarcoma near cartilage (H&E; x40).

**Figure 4:**
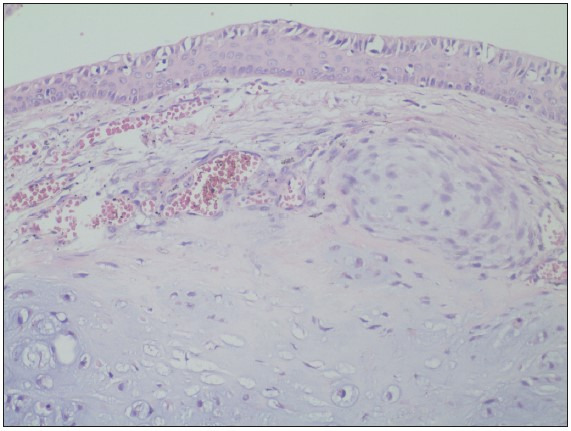
Chondrosarcoma under the respiratory epithelium (H&E; x200).

As a result of these examinations, the patient applied to the Otorhinolaryngology Diseases clinic of Abant Izzet Baysal University Izzet Baysal Training and Research Hospital. The CT showed a subglottic mass compatible with chondrosarcoma arising from the right thyroid cartilage. On axial and coronal MRI images, a malignant mass invading the thyroid, arytenoid and cricoid cartilages with heterogeneous contrast enhancement is observed. (Figure 1) On thorax CT, nonspecific calcific nodules were detected in the lung. No pathology was detected in the abdominal ultrasonography in other examinations. The patient underwent total laryngectomy by the otolaryngology department. In the macroscopy of the laryngectomy material sent to the pathology laboratory, there was a 3x2 cm tumor with a polypoid extension to the lumen from the bottom of the right vocal cord (Figure 2). In the sections made in the larynx, the tumor was located in the thyroid cartilage (Figure 2). Microscopy revealed a tumor with a chondroid matrix located in the thyroid cartilage. Binuclear and multinuclear chondrocytes were observed in the tumor lacunae, and nuclear pleomorphism and hyperchromia were noted in the cells (Figure 3 Figure 4). Mitosis was observed in atypical cells. There were ossification areas in the tumor but no lymphovascular invasion or necrosis was observed. The tumor was found to be invading the posterior muscle fibers. Tumor metastasis was not observed in three dissected lymph nodes. The lesion was been reported as grade 2 chondrosarcoma with these histomorphological findings.

The case with pathological stage 4a received radiotherapy. The patient was followed up for 2 years. He is presently without any sign of recurrence.

## DISCUSSION

Although the etiology of laryngeal chondrosarcoma is not clearly understood, radiation exposure, teflon injection, and disorganized ossification are important risk factors (1,8). There are theories that ischemic changes in the chondroma and chronic inflammation are also factors (9). It is thought to be related to mechanical stress and smoking (5,9).

Although symptoms vary according to the localization of the tumor, dyspnea and airway obstruction and a painless mass may be observed in subglottic lesions (6).

It is difficult to estimate the true incidence of laryngeal chondrosarcoma, as there have been cases of low-grade chondrosarcoma diagnosed in the past as chondroma (9).

The average age at the time of diagnosis is 60-64 years and it is more common in men (6,8). It is most commonly seen in the posterior laminate of the cricoid and then in the thyroid cartilage (9). Our case was an male patient who was older than generally reported in the literature and the lesion was located in the thyroid cartilage.

A 61-year-old patient reported by Moerman et al. had a low-grade chondrosarcoma located in the anterior part of the thyroid cartilage (9). Sauter et al. reported chondrosarcoma developing in two male patients, 93 and 66 years old (5). Two cases reported by Wang et al. were located in the cricoid and 2 cm in diameter, and consisted of low-grade chondrosarcoma (6).

All of the 6 cases by Buda et al. consisted of male patients (8). In addition to various surgical procedures, two patients received radiotherapy and one patient received chemotherapy (8).

One of two cases of Nao et al. was diagnosed with low-grade chondrosarcoma located in the thyroid cartilage (7).

Oliveira et al. presented 6 cases by reviewing the 10-year larynx chondrosarcoma cases (3). Five of these cases were located in the cricoid cartilage and only one was in the thyroid cartilage (3). Five of the patients underwent total laryngectomy (3). A 56-year-old woman with grade 2 tumor located in the thyroid cartilage underwent excision of the left part of the thyroid cartilage (3) .

The diagnosis of low-grade chondrosarcoma can be delayed as it is clinically a slow lesion (7).

On endoscopic examination, it can be observed as a submucosal mass under smooth-looking mucosa, especially in the posterior region, as well as with movement restriction in the vocal cord (3).

Typical finding in CT is hypodense, well-cartied mass with limited calcification (8). MRI is more useful in showing paralaryngeal spread to the tissues (8).

Expansile lesions with popcorn-style calcifications on MRI or CT are important in the diagnosis (5,9,10). The center of the cartilage where the tumor is located has a hypodense appearance (7). MRI examinations show hypointense lesions on T1-weighted sections and hyperintense lesions on T2-weighted sections (4,7). Imaging methods may not be made clear with the diagnosis of chondrosarcoma, and 62% of chondrosarcoma cases may be associated with chondroma (10).

Chondrosarcoma is macroscopically flat, in the form of a lobular mass, and has a translucent appearance (8,10). Tumor size is not related to prognosis, although it can be between 1 and 10 cm in size (10). Deep endoscopic biopsy and histopathological examination are required for a definite diagnosis (4).

In the diagnosis of chondrosarcoma, histological criteria such as cell and nucleus size irregularities, excessive number of cells, hyperchromia, and chromatin aggregation in single or multiple chondrocytes, which were defined by Lichtenstein and Jaff in 1943, are used (5,9). Although the criteria in larynx chondrosarcoma are not fully defined, high cellularity, nuclear pleomorphism, and invasion to neighboring structures are accepted as histological diagnostic criteria. In particular, invasion into cartilage tissue contributes to the diagnosis of chondrosarcoma instead of chondroma. For this reason, it is advocated that all material should be examined microscopically (9).

In the case reported by Ghatak et al., a 42-year-old male patient was diagnosed with the biopsy of a 2 cm diameter mass with an arytenoid location, and a low-grade chondrosarcoma was diagnosed after the operation (2).

Laryngeal chondrosarcoma is graded histologically as grade 1, grade 2, and grade 3 (8). Most laryngeal chondrosarcoma cases (80%) have a lower grade compared to other organ sites (8).

Grade 1 chondrosarcomas are tumors with a good prognosis, and the differential diagnosis with chondroma is clinically, radiologically and pathologically difficult (7). In cases of suspected malignancy, chondrosarcoma should be considered in tumors over 2 cm (7).

In a review by Chin et al., 163 articles were reviewed among articles published over 66 years (1). The average age of 513 patients was 62.5 years, and the male to female ratio was 3/1. The most common site was the cricoid cartilage (333 cases, 56.3%), and the second site was the thyroid cartilage (68 cases, 11.5%) (1). Tumor sizes were an average of 3.7 cm and the most common was histological grade 1 (289 cases, 67.8%) (1).

Histologically, nuclear atypia, increased cellularity, and invasion to neighboring structures are important in the diagnosis (1). Excessive sampling is required to clearly evaluate the separation of chondroma and chondrosarcoma (1). Chondromas are tumors with a homogeneous lobular growth pattern without hypocellular prominent atypia (6). Chondromas are smaller than chondrosarcomas (3). Chondromas consist of small mononuclear chondrocytes with low cellularity. Hyperchromia, mitosis and necrosis are not observed in the chondrocytes. Grade 1 chondrosarcomas contain binuclear and multinuclear chondrocytes but do not show mitosis. Grade 2 chondrosarcomas show increased cellularity and rare mitosis. Grade 3 chondrosarcomas are characterized by chondrocytes with a multinuclear appearance with increased mitotic activity. The presence of the myxoid matrix is also associated with more aggressive behavior. The rate of metastasis increases with increasing grade and is 10% in grade 2 chondrosarcoma (10). Our case was grade 2 and no metastasis was observed.

Chondrosarcoma should be differentiated from laryngeal nodular chondromalacia, fibrosarcoma and osteosarcoma, as well as chondroma (3,4). The presence of lymph node metastasis and local invasion suggest a high-grade tumor (4).

Although the treatment protocols vary, the main treatment protocol is surgery (8). In low-grade tumors, while preventing laryngeal function treatments such as CO2 laser ablation and hemilaryngectomy, total laryngectomy is performed in patients with large tumors and high grade (1,8,10). Since the chondrosarcoma is not sensitive to radiation, the place of radiation in the treatment is limited (7). Radiotherapy can be added to the treatment in case of recurrence, aggressive tumors, and inoperable tumors (3).

Laryngeal chondrosarcoma has a better prognosis than non-larynx localized chondrosarcoma and can be detected at an earlier stage (3).

Laryngeal chondrosarcoma has a good prognosis and rarely metastasizes (8). Metastases are to the cervical lymph nodes, lung, bone, and liver (2,4). However, recurrence can be seen in 35-40% (8). Recurrence can be seen a few months or a few years after treatment in relation to incomplete resection and tumor grade (9). Tumor-related death is associated with uncontrolled tumor growth, recurrence, the presence of aggressive tumors, and tumor invasion into vital structures (5). The prognosis is better for early stage laryngeal chondosarcomas (11).

Since laryngeal chondrosarcoma may relapse in the late period, it requires long-term follow-up (3).

Laryngeal chondrosarcoma is a rare, low-grade tumor with a good prognosis. This case was a chondrosarcoma located in the thyroid cartilage, which is a rare site. Although clinical and radiological findings are important in the diagnosis, the definite diagnosis is based on the pathological examination. It is especially important to differentiate the lesion from chondromas. Adequate macroscopic sampling to evaluate the tumor site and invasion areas clearly after macroscopic evaluation is important in the correct diagnosis.
